# Reconstructing diploid 3D chromatin structures from single cell Hi-C data with a polymer-based approach

**DOI:** 10.3389/fbinf.2023.1284484

**Published:** 2023-12-11

**Authors:** Jan Rothörl, Maarten A. Brems, Tim J. Stevens, Peter Virnau

**Affiliations:** ^1^ Institute of Physics, Johannes Gutenberg-Universität Mainz, Mainz, Germany; ^2^ MRC Laboratory of Molecular Biology, Cambridge, United Kingdom

**Keywords:** chromatin, Hi-C, 3D structure, polymers, diploid cells, interphase, molecular dynamics

## Abstract

Detailed understanding of the 3D structure of chromatin is a key ingredient to investigate a variety of processes inside the cell. Since direct methods to experimentally ascertain these structures lack the desired spatial fidelity, computational inference methods based on single cell Hi-C data have gained significant interest. Here, we develop a progressive simulation protocol to iteratively improve the resolution of predicted interphase structures by maximum-likelihood association of ambiguous Hi-C contacts using lower-resolution predictions. Compared to state-of-the-art methods, our procedure is not limited to haploid cell data and allows us to reach a resolution of up to 5,000 base pairs per bead. High resolution chromatin models grant access to a multitude of structural phenomena. Exemplarily, we verify the formation of chromosome territories and holes near aggregated chromocenters as well as the inversion of the CpG content for rod photoreceptor cells.

## 1 Introduction

Reconstruction of 3D chromatin conformations is a promising approach to improve our understanding of processes in the cell. While coarse information about the existence of chromosome territories has already been obtained with different methods (Cremer et al., 1993; Cremer and Cremer, 2001; Bolzer et al., 2005; Branco and Pombo, 2006), chromosome conformation capture methods like Hi-C (Lieberman-Aiden et al., 2009) provide more detailed and accurate information about the organization of chromatin. These results can, e.g., be used to improve the understanding of the cell cycle (Naumova et al., 2013), gene regulation (Cremer and Cremer, 2001) and differentiation of cell types (Dixon et al., 2015).

While initially Hi-C was used for bulk data due to the larger amount of accessible contacts (Stefano et al., 2013; Pierro et al., 2016; Stefano et al., 2016; Dudchenko et al., 2017), more recent approaches use single cell Hi-C (Nagano et al., 2013; Flyamer et al., 2017; Nagano et al., 2017; Ramani et al., 2017; Siebert et al., 2017; Stevens et al., 2017; Tan et al., 2018; Oluwadare et al., 2019) to determine chromatin structure as it accounts for cell-to-cell differences (Ramani et al., 2017). Current models may analyze single-cell Hi-C data without creating 3D structures (Flyamer et al., 2017; Ramani et al., 2017; Ing-Simmons et al., 2021; Zhang et al., 2022), reconstruct complete chromatin structures up to a resolution of 20,000 base pairs per bead (bp) (Tan et al., 2018) or lower (Nagano et al., 2013; Nagano et al., 2017; Stevens et al., 2017; Wettermann et al., 2020) or resolve specific regions of chromatin at higher resolutions (Huang et al., 2020). This is done using various approaches like minimization of bead-spring polymer models with simulated annealing (Nagano et al., 2013; Nagano et al., 2017; Stevens et al., 2017), manifold based optimization (Paulsen et al., 2015) or Bayesian inference (Rosenthal et al., 2019).

In this work, we build upon a polymer-based minimization protocol for low-resolution haploid cells (Wettermann et al., 2020), which allows us to resolve 3D structures of diploid cells up to a maximum of 5 kbp resolution. Essentially, the resolution of the model is successively increased by assigning ambiguous Hi-C contacts based on emerging lower resolution structures. Our procedure is tested for consistency of contact lists and chromosome territories. We also re-analyze the spatial distribution of specific parts of DNA like CpG sites (Tan et al., 2018), which play an important role in DNA transcription (Fatemi et al., 2005).

## 2 Materials and methods

### 2.1 Polymer-based chromatin model and basic minimization procedure

Individual chromosomes are modeled as coarse-grained bead-spring polymers consisting of spherical beads connected by harmonic springs (Wettermann et al., 2020). One bead represents between 5,000 and 5,000,000 base pairs depending on the chosen resolution. The specific shape for the analyzed cell is enforced with a second harmonic spring potential connecting non-adjacent beads in contact with each other according to experimental single cell Hi-C matrices. Excluded volume required to enforce non-contacts is implemented via a Gaussian potential:

$$V_{\text{Bond/Contact}}\left(r\right)=\frac{1}{2}k_{\text{b/c}}{\left(r-r_{\text{0,b/c}}\right)}^{2}$$ (1)

$$V_{\text{Gauss}}\left(r\right)=\left\{\begin{array}{cc} \varepsilon \exp\left(-\frac{1}{2}{\left(\frac{r}{\sigma }\right)}^{2}\right)\; & r<r_{\text{cut}}\\ 0\; & r\geq r_{\text{cut}}. \end{array}\right.$$ (2)

Here, *r* refers to the distance between two beads. *r*_0,b_ = 1 and *r*_0,c_ = 1.5 are preferred distances at which bond or contact energy terms are minimal. While these choices are somewhat arbitrary, differences in *r*_0_ ensure that the degeneracy of the ground state of the model is reduced, i.e., each minimization procedure results in a very similar structure. Likewise, final values for *k*_b_ and *k*_c_ are large (2000) to ensure small variations of bond and contact distances. Excluded volume is characterized by a width *σ* and a scale *ϵ* and acts up to a cutoff distance *r*_cut_ chosen to be around 1 percent of the maximum of the potential to enforce numerical stability during energy minimization. If not mentioned otherwise, all numbers are given in simulation units.

**FIGURE 1:**
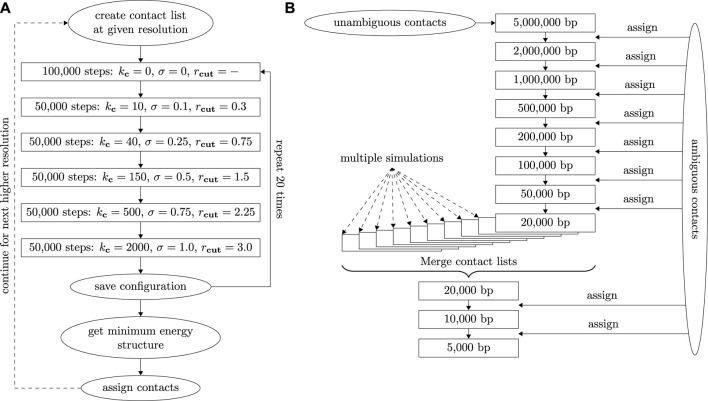
**(A)** Diagram depicting the simulation process at a given resolution including contact assignment and changes of interaction potentials. **(B)** Diagram depicting the contact assignment process with adaptive resolution starting at 5 Mbp. After the first simulation at 20 kbp resolution, contacts from 10 independent runs are merged to get a common contact list.

**FIGURE 2:**
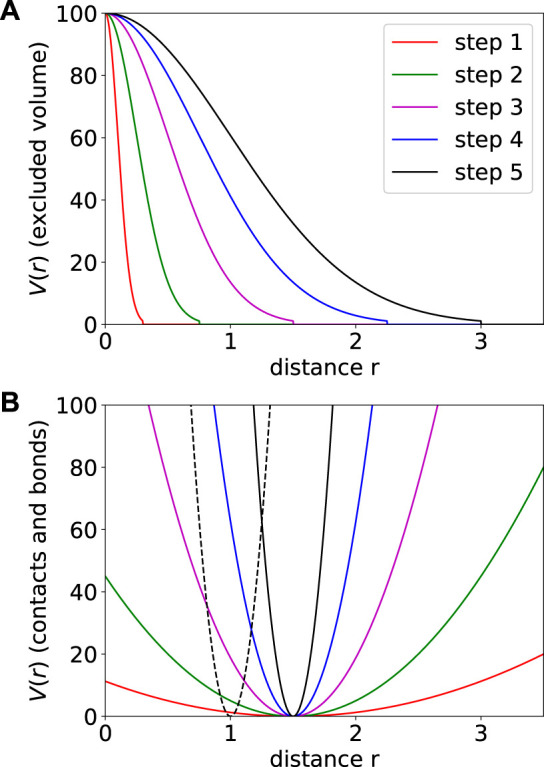
Gradually increasing excluded volume **(A)** and contact potentials **(B)** potentials used in the course of the minimization procedure. Potentials drawn in the same color in **(A)** and **(B)** belong to the same step. The excluded volume is increased by increasing the width *σ* while for the contact potential *k*_*c*_ is increased. The (fixed) bond potential (*k*_*b*_ = 2000) is indicated by a black dotted line in **(B)**.

A Molecular Dynamics minimization run starts by placing beads into a small cube of size 10^3^. Beads are connected by the bond potential to form individual chromosomes and equilibrated for a few time steps. Afterwards, contact potentials and excluded volume interactions are enforced and gradually increased in five steps by varying *k*_c_ and *σ*, respectively. Throughout this process, intra- and inter-chain bond crossings occur and enable conformational and topological rearrangement of the chromatin structure. Figure 1A provides the complete protocol including parameters and time steps for each phase - potentials are visualized in Figure 2. The final potentials are the same as in our previous work (Wettermann et al., 2020). Intermediate steps are chosen to ensure smooth adjustments from zero to full potentials. Typical 3D structures at different stages of the minimization can be found in Supplementary Figure S2. After a final structure has been created, bond and contact potentials are reset to zero, the structure collapses, and the procedure can start all over again to generate independent conformations. In previous work (Wettermann et al., 2020), we have shown that a similar protocol always leads to similar structures, which fulfill bond and contact requirements and exhibit pronounced chromosome territories. A further verification of this algorithm by using it to reproduce polymer globules (Virnau et al., 2005) is given in Supplementary Material. Note, however, that mirror images of structures may occur in the process as information on the chirality of the whole chromatin structure is not encoded in the contact matrix. In contrast, the relative chirality of individual chromosomes or regions of chromosomes is encoded in the long-range intra-chromosomal and inter-chromosomal contacts in the contact matrix and therefore not arbitrary. Regions lacking these contacts could, however, have ambiguous local chirality leading to a larger uncertainty of the ensemble of different structures. The energy minimization procedure is loosely inspired from methods to generate starting conformations of polymer melts (Auhl et al., 2003). Simulations are performed on GPUs using the general Molecular Dynamics simulation toolkit HOOMD-blue (Anderson et al., 2008; Glaser et al., 2015) with a Langevin thermostat (damping constant *γ* = 1, temperature *k*_*B*_ *T* = 1) and a time step of 0.001.

### 2.2 Mapping of contacts and assignment of ambiguous contacts

In a diploid structure, single cell Hi-C contacts can often not be assigned unambiguously to the individual chromosomes in homologous pairs. In the worst case, four assignments are conceivable corresponding to the four permutations of two chromosome pairs in question. Here, we perform this assignment subsequently by creating energy-minimized structures with increasing resolutions and using these structures for assignment of ambiguous contacts for higher resolution runs. At low resolutions we aim to have around one unambiguous contact per bead. This ensures that the larger scale chromosome geometry and packing is intact, and hence we can estimate for a given contact which permutations of homologous chromosomes are compatible with the emerging structure. A contact which is inconsistent with a low resolution structure is likely also inconsistent at higher resolution even though our procedure in principle allows for this possibility.

Contacts are mapped to simulation beads by determining which beads contain contact sites. If the two partners of a contact are located on the same bead or in beads already connected by a bond-potential, the contact is omitted for the given resolution but can still be relevant for higher resolutions. Duplicate contacts are also omitted leading to a fixed contact potential amplitude irrespective of the number of contacts.

We start a first minimization run at a resolution of 5 Mbp resulting in a coarse structure which can be created using only unambiguous contacts. The resolution is then increased up to 20 kbp in eight steps (5M, 2M, 1M, 500k, 200k, 100k, 50k, 20k). After each step, we assign contacts by comparing the distance of all potential contact pairs. This process of contact assignment at increasing resolutions is illustrated in a diagram shown in Figure 1B. A contact is assigned if one assignment option has a distance of less than 3.0 in the minimized structure as the distribution of contract distances decreases significantly for larger values. At the same time the potential contact distances of all other assignment options in the structure need to exceed a value of 5.0 which is incompatible with the distribution of contact distances. If a contact distance becomes larger than 3.0 in the minimized structure, this contact will not be used in the next simulation run (even if it was unambiguous to begin with) and its assignment to specific chromosomes will not be saved. After this run, the contact can, however, be assigned again using the above criteria. This removal option accounts for the uncertainty of the assignment as it allows for correction of assignment and potential Hi-C mapping errors. The reliability of the removal of incorrect assignments was further probed by performing simulations with additional randomly placed contacts (5%) at 100 kbp. In this test, our procedure was able to remove around 90% of the spurious contacts. The distribution of regular and spurious contacts is shown in Supplementary Figure S3.

A resolution of 20 kbp is the highest resolution which can be obtained reliably in a single series of minimization runs, which coincides with the highest resolution in Tan et al. (2018) on which our structures are based. In order to increase resolution further, we merge contact lists from 10 independent minimizations series (each starting at 5 Mbp) to increase the number of contacts: Incompatible contacts which are assigned inconsistently across different runs are discarded as it is not possible to reliably assign them to one specific pair of contact partners. In the case of the cell gm12878_17 (Tan et al., 2018) presented here, e.g., each individual run gave roughly 766,000 contacts. After the merge, a total of 1.01 million contacts were assigned while about 43,000 contact pairs from individual simulations were discarded. Note that these numbers include duplicates at a given resolution and contacts within a bead. The actual number of contacts used for the merged 20 kbp structure is around 499,000. Based on this new contact list, another minimization run is performed at 20 kbp before the resolution is increased further to 10 and finally 5 kbp. The total simulation cycle takes between 2 days and a week on a single GPU depending on the amount of base pairs and contacts and the computational power of the specific GPU used. (In this work we mostly used Nvidia RTX 2070 cards and Nvidia Tesla V100 SXM2 for the higher resolution structures as the latter required more video memory). The concrete protocol should also only serve as a guideline and may need to be adjusted depending on the quality of the available data. Specifically, the initial resolution can be higher if more unambiguous contacts are known. Similarly, the final resolution is limited by the total number of contacts. In comparison to our previous studies on haploid cells (Wettermann et al., 2020), here we had to increase the amount of steps in which the interaction potentials were incremented to account for larger cells and higher resolutions to ensure that the simulation program can still thermally equilibrate the conformations appropriately.

### 2.3 Removal of edge segments without contacts and reflection of mirror images

Typical experimental contact lists contain fairly large parts with only few Hi-C-contacts due to repetitive sequences, so some parts of the chromosomes are not connected to the main structure but form “arms”. These minimization artifacts are located outside the main structure and have different positions in each simulation run. Here, outliers are defined by counting the number of contacts in a 0.5 megabasepair neighborhood of each bead. As suggested by Tan et al. (2018), the 6% of the beads with the lowest number of nearby contacts are removed from the final structures for visualization and analysis purposes. This exclusion could alternatively be performed by removing beads with high RMSD. This approach would lead to a similar result as beads with few contacts are typically less located.

Finally, we need to map mirror-inverted structures onto each other. These mirrored structures occur as information on chirality cannot be deduced from contact lists alone, i.e., a structure and its mirror image are both compatible with the same contact list (Wettermann et al., 2020). As a measure of chirality we use the sign of the triple product of the center of mass positions of the first three chromosomes after translating the entire structure into its total center of mass

$$\text{Chir}\left({\vec{R}}^{\left(1\right)},{\vec{R}}^{\left(2\right)},{\vec{R}}^{\left(3\right)}\right)=\text{sgn}\left(\det\left(\begin{array}{ccc} R_{x}^{\left(1\right)} & R_{y}^{\left(1\right)} & R_{z}^{\left(1\right)}\\ R_{x}^{\left(2\right)} & R_{y}^{\left(2\right)} & R_{z}^{\left(2\right)}\\ R_{x}^{\left(3\right)} & R_{y}^{\left(3\right)} & R_{z}^{\left(3\right)} \end{array}\right)\right),$$ (3)

where sgn is the sign function, det the determinant and $R_{j}^{\left(i\right)}$ the *j*th component of the center of mass vector of the *i*th chromosome. In principle, one could also use a singular value decomposition of the same matrix to detect different chiralities. The choice of the first three chromosomes is arbitrary but assuming that the simulated structures have the same chromosome territories at the same place, any choice allows for the same chirality correction. The validity of this assumption has been checked on some samples for 100 kbp and higher resolutions. This result is then used to mirror all structures with Chir = −1 such that all structures have the same chirality.

## 3 Results

### 3.1 Cell structures at different resolutions and quality assessment

**FIGURE 3:**
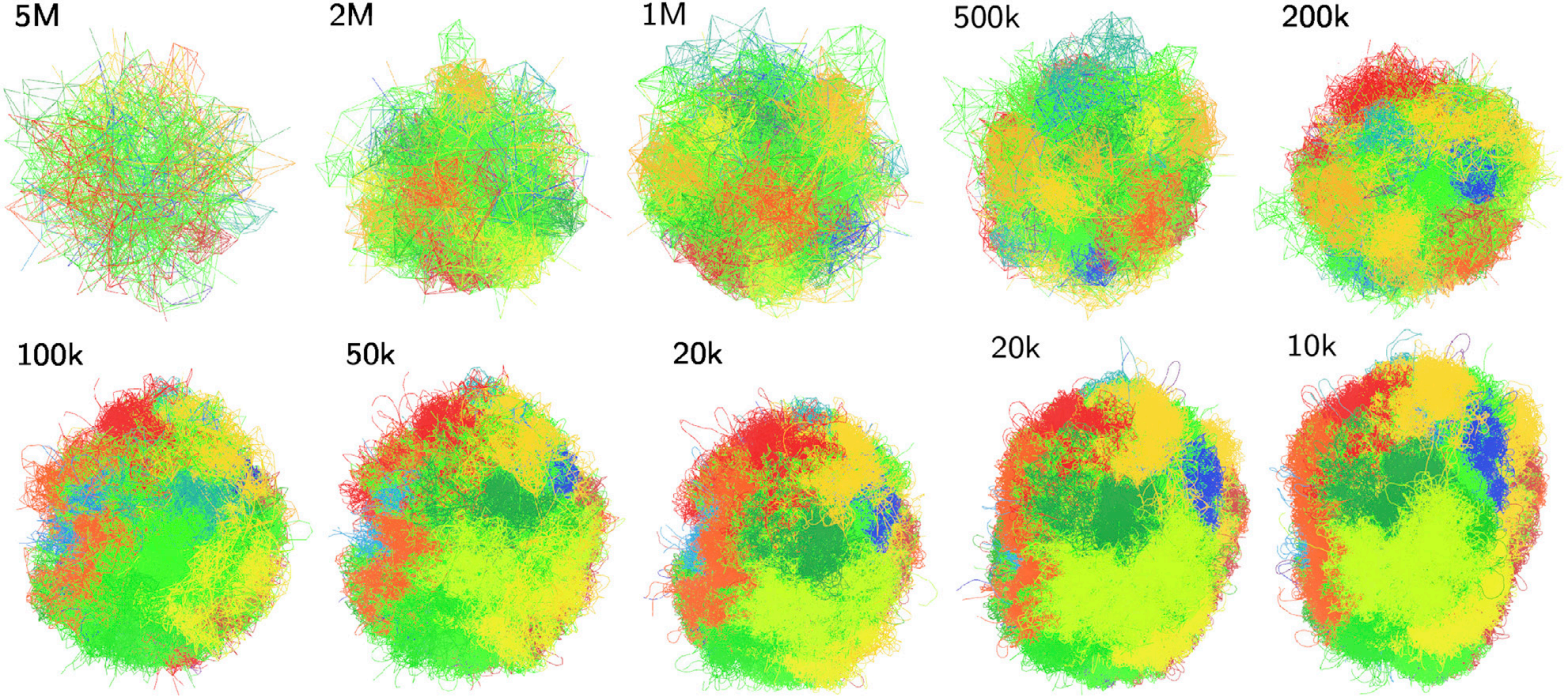
Structures of cell gm12878_17 obtained for all resolutions between 5,000,000 and 10,000 bp: The last two structures are using the combined contact list. Structures have been scaled to a similar overall size, rotated and mirrored to allow for a meaningful comparison.

In the following we visualize our approach and gauge the quality of resulting chromatin structures using an experimental data set for single diploid human cells from Tan et al. (2018). Representative structures at various resolutions are shown in Figure 3. At low resolutions chromosome territories are still not well-developed. Meaningful structures start at a resolution of around 100 kbp (*F*), and exhibit prominent chromosome territories upon increasing the resolution further. The root mean square deviation (RMSD) is employed as a measure for the quality of created structures. Its value between structure *j* and a structure with mean coordinates is defined as

$${\text{RMSD}}_{j}=\sqrt{\frac{1}{N}\overset{N}{\underset{i=1}{\sum }}{\left(\vec{r_{i}}\left(j\right)-\left\langle \vec{r_{i}}\right\rangle \right)}^{2}}.$$ (4)

**FIGURE 4:**
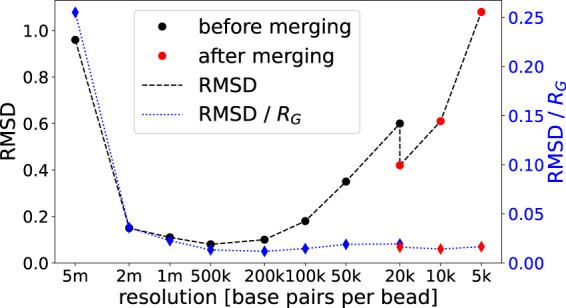
Average RMSD values of 20 different structures per resolution obtained from one specific run compared to respective structures with mean coordinates. Lines are added for readability.

High values of the RMSD indicate that structures from different minimization runs (using the same list of contacts) differ significantly and therefore the structures are of low precision. RMSD values for all resolutions are presented in Figure 4. While structures at the coarsest resolution exhibit a rather large RMSD (which in part can be explained by problems arising from our chirality transformation for very coarse structures), the latter drops and stays low up to 100 kbp before rising again due to the reduced number of contacts per bead. Note that the RMSD is given in simulation units and therefore the same RMSD for a higher resolution indicates a smaller relative deviation of the chromatin structure (as beads shrink). The data merge at 20 kbp (which is the highest resolution presented in Tan et al. (2018)) results in a significant drop of the RMSD which enables us to increase resolution further by a factor of two or even four. Overall, structures emerging from our procedure yield similar results as the ones provided by Tan et al. (2018). The RMSD of the two 20 kbp structures with the lowest energy resulting from completely independent runs starting at 5 Mbp resolution is 0.96 (after the merge) as opposed to 1.23 in Tan et al. (2018) after correcting for chirality.

**FIGURE 5:**
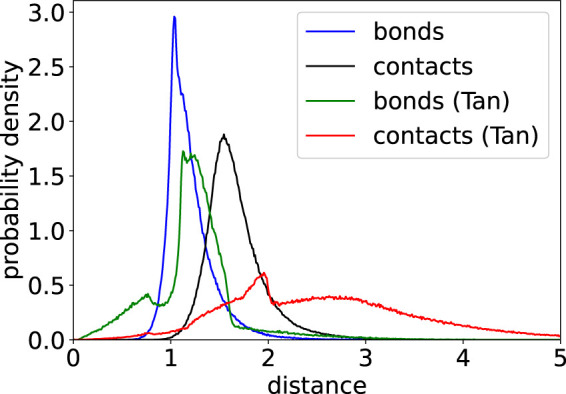
Probability density of bond and contact lengths for a 20 kbp structure from our minimization protocol and minimizations performed by Tan et al. (2018).

Bond and contact length distributions of 3D structures indicate how well simulation results agree with a given list of contacts. A perfect structure would exhibit narrow peaks of these distributions near the potential minima, which are at 1.0 for bonds and at 1.5 for contacts in this work. However, limitations of our model such as potential assignment errors and the incompleteness of the underlying experimental contact matrix as well as potential mapping errors lead to broader distributions with slightly overstretched bonds and contacts as shown in Figure 5. In comparison, results of Tan et al. (2018) show a more complex behavior with several peaks and a somewhat broader distribution.

**FIGURE 6:**
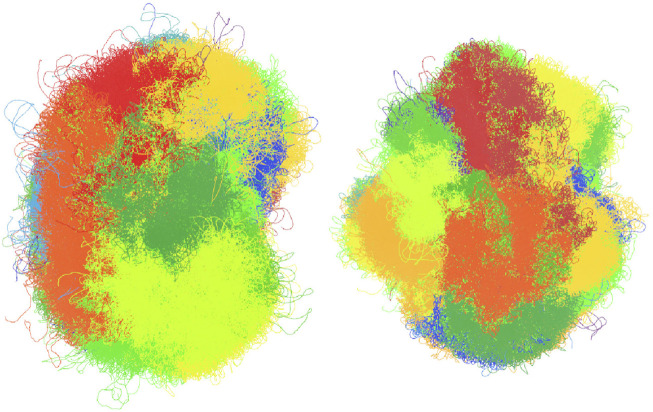
Two different perspectives on the chromatin structure of cell gm12878_17 (Tan et al., 2018) at a resolution of 5 kbp. The two images have the same relative size.

The final step of the simulation protocol yields structures at a resolution of 5 kbp. One such structure is presented from two perspectives in Figure 6. Chromosome territories are clearly visible for all chromosomes and the overall shape of the 3D structure is rather prolate than spherical and somewhat rougher compared to lower resolution structures, which indicates that the resolution should not be increased further (in agreement with our observations of RMSDs). Further cells from the same data set are displayed in Supplementary Figure S4. In Supplementary Figure S5 we also display a contact matrix based on the final 5 kbp structure shown above. Recorded Hi-C contacts roughly account for 1.5% of the close beads pairs observed in the final structure (defined as distances smaller than 3) which lies in the expected range (Stevens et al., 2017).

### 3.2 Rod photoreceptor cells

Our procedure was also adapted to rod photoreceptor cells of mice from Tan et al. (2019). As less Hi-C contacts were available for this data set, the protocol was modified to only include resolutions up to 20 kbp and exclude merging of contact lists. Additionally, the lowest simulated resolution was 500 kbp because the set contains a larger fraction of unambiguous contacts.

**FIGURE 7:**
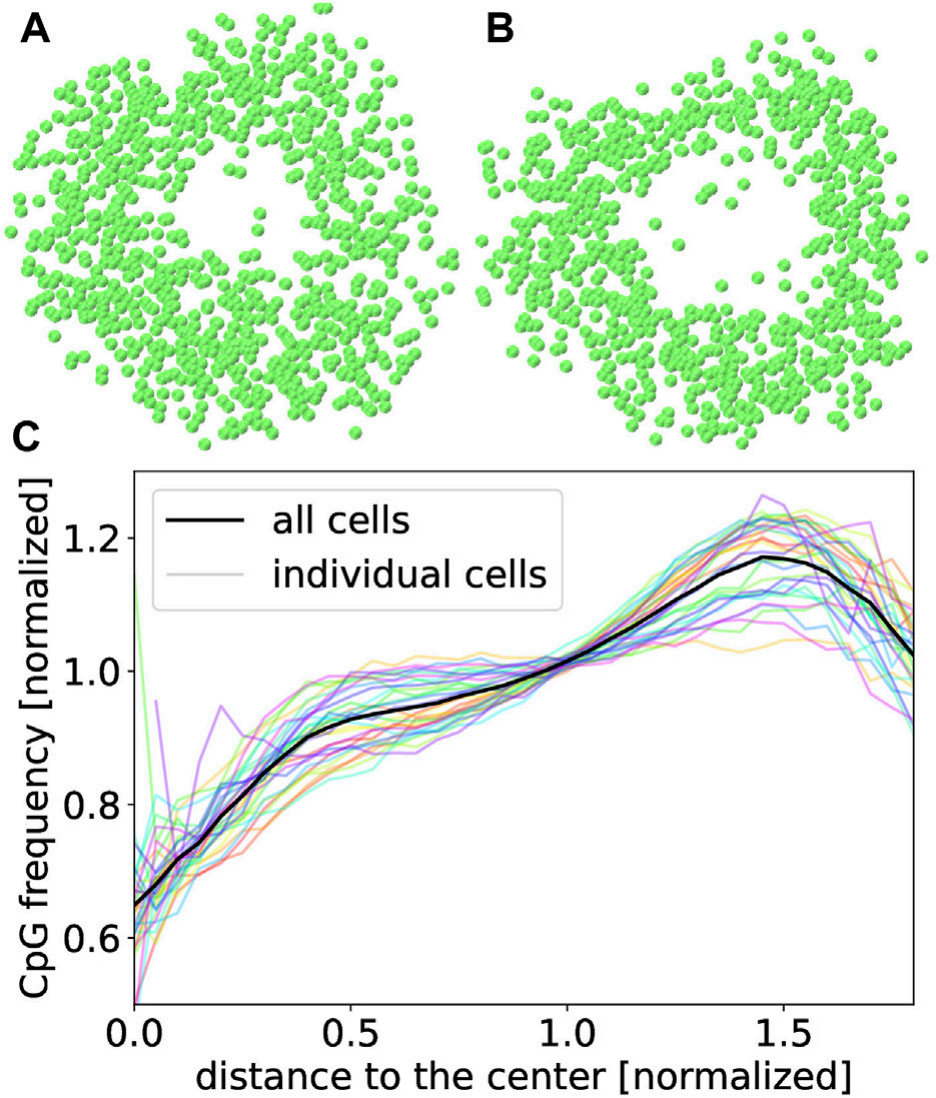
Cross-sections through the center of 20 kbp structures of cell 170 from Tan et al. (2019) **(A)** and from this work **(B)**. Both cuts are done along the same plane. **(C)** CpG density averaged over 30 different male young adult rod photoreceptor cells from Tan et al. (2019) at a resolution of 20 kbp. The transparent lines show the CpG density of each individual cell.

Resulting 3D structures contain a clearly visible hole for most rod photoreceptor cells as shown in Figure 7. Holes resulting from our procedure (B) tend to be somewhat larger than in structures published by Tan et al. (2019) (A) which can likely be explained by the employment of spherical confinement in (A) while our minimizations were unconstrained.

Another special property of rod photoreceptor cells is the inverted CpG density distribution. While most cells have the highest density of CpG sites near their center, rod photoreceptors are assumed to have the highest value in the periphery (Solovei et al., 2009; Tan et al., 2019). This inversion was found for all 30 cells we have analyzed as shown in Figure 7.

## 4 Discussion

We developed a computational scheme based on Molecular Dynamics simulations at multiple resolutions and a bead-spring model for DNA which allows for assignment of ambiguous single cell Hi-C contacts applying a structure-based approach. As a result, robust single-cell 3D structures of interphase chromatin in diploid cells could be simulated at resolutions of up to 5,000 base pairs per bead improving upon previously achievable resolutions based on the same data (Tan et al., 2018). Computational effort to recreate 3D structures at a resolution of 20 kbp only amounts to a few hours on a single GPU, while resolutions of 5 kbp can be obtained in a few days. Our model and procedure was verified and assessed on two previously published data sets of diploid cells (Tan et al., 2018; 2019). We were also able to reproduce prominent structural features such as CpG inversion in photoreceptor cells while maintaining a high level of structural fidelity as indicated by low RMSDs and rather tight bond and contact distributions. In future research, one could test the reliability and potentially extend our approach to even more challenging systems such as tetraploid cells (Sun et al., 2022) or cells exhibiting chromosome alignment.

## Data Availability

Publicly available datasets were analyzed in this study. This data can be found here: https://www.ncbi.nlm.nih.gov/geo/query/acc.cgi?acc=GSE117874 and https://www.ncbi.nlm.nih.gov/geo/query/acc.cgi?acc=GSE121791. The code required to perform the computer simulations presented here is publicly availible on https://gitlab.rlp.net/3d-diploid-chromatin/simulation-code/.
