# Supplementary material for: Reconstructing diploid 3D chromatin structures from single cell Hi-C data with a polymer-based approach
Source: Front Bioinform. 2023 Dec 11;3:1284484. doi: 10.3389/fbinf.2023.1284484 (PMC10750380; doi:10.3389/fbinf.2023.1284484)
Supplement: Supplementary file 1 [file DataSheet1.PDF]

Supplementary Material

1 VERIFICATION OF STRUCTURE REPRODUCTION ALGORITHM

To further verify the accuracy of the described method, we performed simulations reproducing a generic 3D polymer structure taken from Virnau et al. (2005). This is inspired by Nagano et al. (2013) who verified their approach for 3D structure modelling (of individual chromosomes instead of whole cells) by reproducing Hilbert curves.
Contacts in the original polymer structure are determined by Voronoi tessellation. To reproduce the structure, we used 10% of the contacts which corresponds to approximately 0.5 contacts per bead. Altogether we have generated three sets with different contacts and ten structures for each set. From these structures we have selected the one with the lowest energy for comparison.Monomers of the original model interact via cut and shifted Lennard-Jones (LJ) interactions, and adjacent beads are connected with FENE springs. Typical distances are 0.97 for connected beads and $\sqrt[6]{2}$ for unconnected beads which correspond to the minimum of the LJ+FENE and the LJ potential, respectively. To account for this, we have switched modelling potentials to WCA (LJ potential without attractive part) for non-bonded interactions. For the first two steps we still use a Gaussian potential, however, as a finite maximum force at the beginning is required for computational stability. The simulation protocol is given in Table S1. Bonds and contacts are realized with a harmonic potential with a minimum at 0.97 and $\sqrt[6]{2}$, respectively.
As indicated in Figure S1 reproduced structures (as the one shown on the right) agree well with the original (left). The average RMSD between the lowest energy structures from our three sets (which were derived from different contacts) and the original is only 0.8, while the RMSD for structures from the same set is about 0.6. Two different original structures on the other hand, have an RMSD of 3.5. The radii of gyration are also very similar: 5.8 for the original and 6.1 for the reproduced lowest energy structures.
We also checked for knots using HOMFLYPT polynomials Dabrowski-Tumanski et al. (2020) and noticed that reproduced structures tend to be more knotted than the original structure in line with our observation in Siebert et al. (2017).

step	k_b	k_c	excluded volume	ϵ	σ	r_cut	MD steps
0	2,000	0	None	-	-	-	100,000
1	2,000	10	Gaussian	100	0.1	0.3	50,000
2	2,000	40	Gaussian	100	0.25	0.75	50,000
3	2,000	150	LJ	1	0.5	$0.5 \cdot \sqrt[6]{2}$	50,000
4	2,000	500	LJ	1	0.75	$0.75 \cdot \sqrt[6]{2}$	50,000
5	2,000	2,000	LJ	1	1.0	$1.0 \cdot \sqrt[6]{2}$	50,000

Table S1. Simulation parameters at subsequent stages of the simulation of polymer globules.

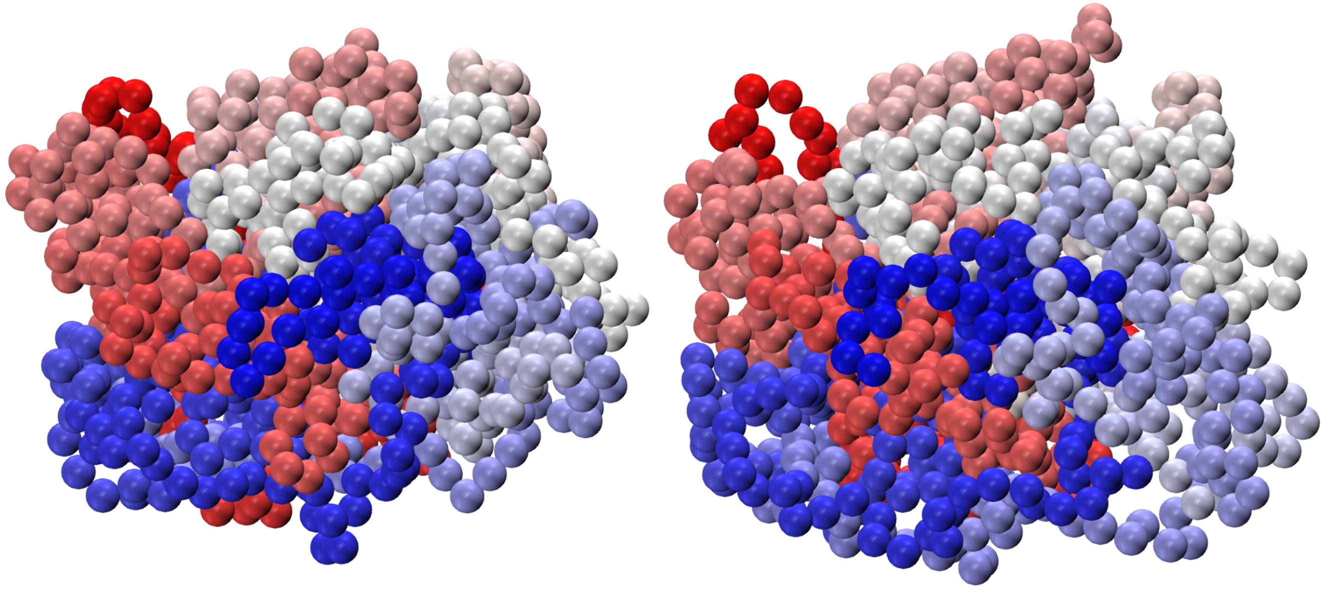

Figure S1. Reproduction of a globular structure with 1000 beads. The left picture shows a structure from Virnau et al. (2005) and the right picture shows a reproduction based on 10 percent of the contacts. Colors are chosen based on the bead index.

2 SUPPLEMENTARY FIGURES

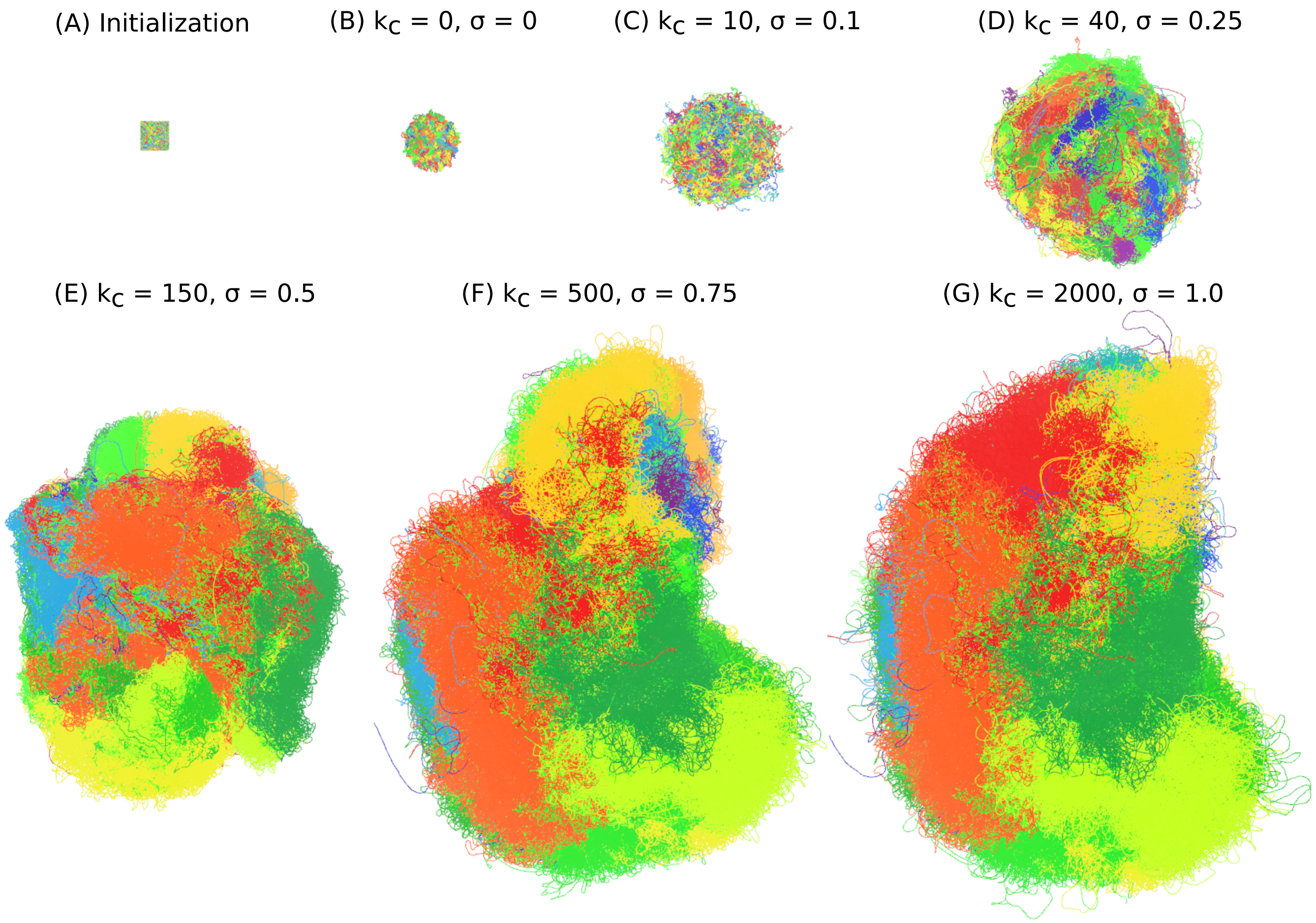

Figure S2. Structures for different values of the excluded volume potential during one step of the simulation for a structure of cell `gm12878_17` Tan et al. (2018) at a resolution of 5 kbp. All structures are displayed in the same relative scale and show a significant increase in size. Individual chromosomes are represented by different colors to aid visualization. The two copies of the same chromosome are represented by the same color. (A) Initial configuration with all particles placed randomly inside a cube. (B) Individual chromosomes are connected via bond potentials ($k_b = 2000$) while contact and excluded volume potentials are still set to zero. (C-G) Configurations are created using the step-wise increased excluded volume and contact potentials as given in table 1 in the manuscript. (G) uses the full potentials.

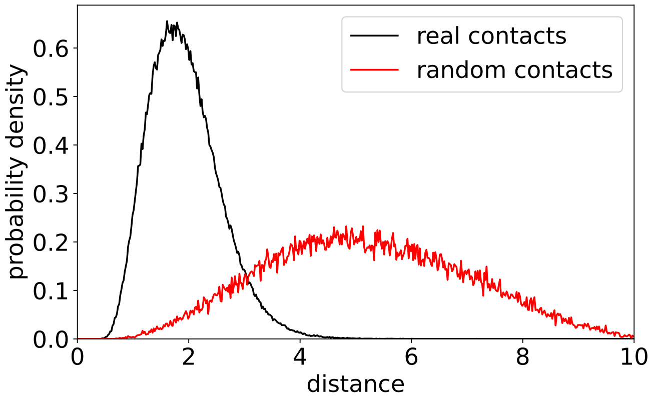

Figure S3. Contact length distributions for a 100 kbp structure calculated with five percent random contacts. The black line shows the real contacts in this figure while the red line shows the random ones. 88.7% of random contacts are farther than 3.0 and would therefore be removed during contact assignment.

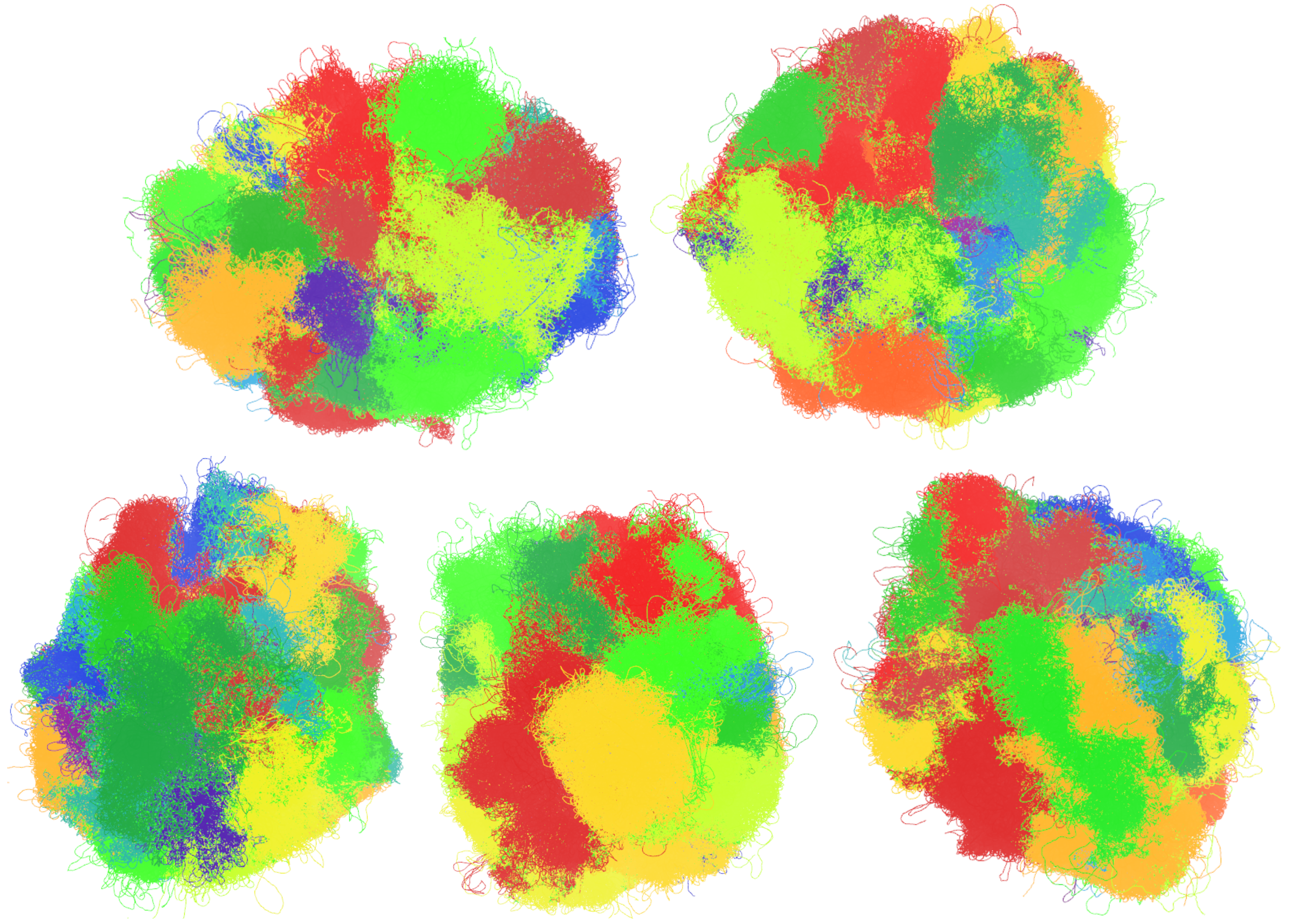

Figure S4. Final structures at a 5 kbp resolution for other cells from `gm12878` Tan et al. (2018). The same chromosomes are colored accordingly in every cell.

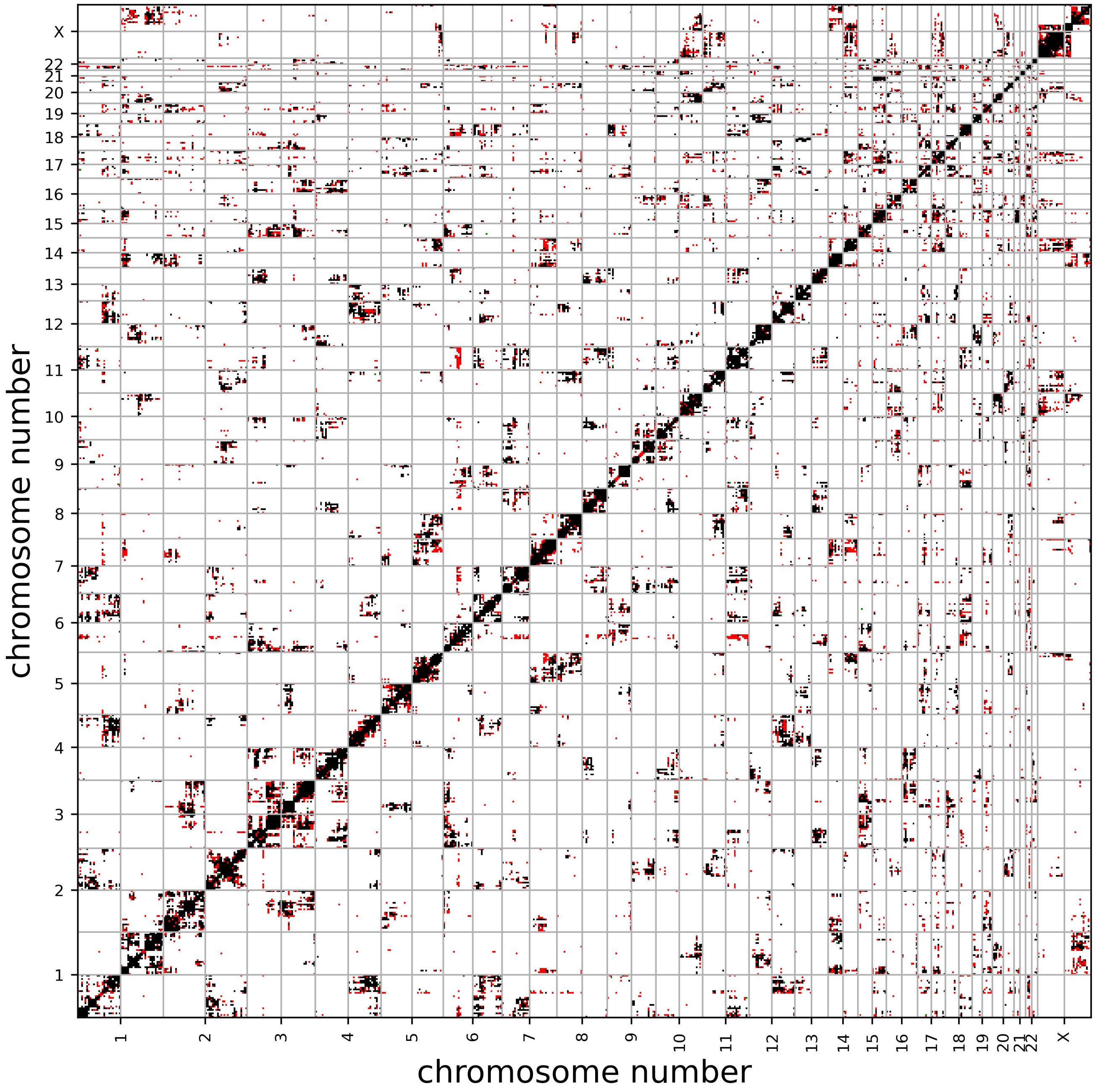

Figure S5. Matrix showing all contacts at a resolution of 1,000,000 base pairs per bin. Black dots are positions that are both, connected by an assigned Hi-C contact and closer than 3.0 in the simulated structure at 5 kbp. Red dots are closer than 3.0 in the 3D structure but not connected by Hi-C contacts. Blue dots are connected by Hi-C contacts, but not close in the 3D structure. These occur rarely and are barely visible.

REFERENCES

Dabrowski-Tumanski, P., Rubach, P., Niemyska, W., Gren, B. A., and Sulkowska, J. I. (2020). Topoly: Python package to analyze topology of polymers. *Briefings in Bioinformatics* 22, bbaa196. doi:10.1093/bib/bbaa196

Nagano, T., Lubling, Y., Stevens, T., Schoenfelder, S., Yaffe, E., Dean, W., et al. (2013). Single-cell Hi-C reveals cell-to-cell variability in chromosome structure. *Nature* 502, 59–64. doi:10.1038/nature12593

Siebert, J. T., Kivel, A. N., Atkinson, L. P., Stevens, T., Laue, E., and Virnau, P. (2017). Are there knots in chromosomes? *Polymers* 9, 2–10. doi:10.3390/polym9080317

Tan, L., Xing, D., Chang, C.-H., Li, H., and Xie, X. S. (2018). Three-dimensional genome structures of single diploid human cells. *Science* 361, 924–928. doi:10.1126/science.aat5641

Virnau, P., Kantor, Y., and Kardar, M. (2005). Knots in globule and coil phases of a model polyethylene. *Journal of the American Chemical Society* 127, 15102–15106. doi:10.1021/ja052438a
